# Development and validation of an interpretable machine learning–based model for predicting carbapenem-resistant *Acinetobacter baumannii* infection in postoperative ICU patients: a retrospective cohort study

**DOI:** 10.3389/fcimb.2026.1788229

**Published:** 2026-03-25

**Authors:** Yan Gao, Guangxin Gu, Ruiwen Wang, Chen Jia, Feng Zhao, Dan Cao, Xin Jin, Xiaoyuan Ma, Yu Wang, Xueyu Li

**Affiliations:** 1 Department of Disease Prevention and Control, General Hospital of Northern Theater Command, Shenyang, China; 2 Key Laboratory of Environmental Stress and Chronic Disease Control & Prevention, China Medical University, Ministry of Education, Shenyang, China; 3 Department of Epidemiology, School of Public Health, China Medical University, Shenyang, China; 4 Department of Orthopedics, General Hospital of Northern Theater Command, Shenyang, China

**Keywords:** carbapenem exposure, carbapenem-resistant *Acinetobacter baumannii*, central venous catheterization, ICU length of stay, los, machine learning models, mechanical ventilation, postoperative ICU patients

## Abstract

**Background:**

Carbapenem-resistant *Acinetobacter baumannii* (CRAB) is a major cause of healthcare-associated infections and is associated with poor outcomes in intensive care units, particularly among postoperative patients. However, predictive tools for early identification of high-risk postoperative intensive care units (ICU) patients remain scarce.

**Methods:**

We conducted a retrospective cohort study including 2,195 postoperative ICU patients. Clinically available demographic, treatment-related, and laboratory variables were used to develop eight machine learning models. Feature selection was performed using Boruta, and model interpretability was enhanced using Shapley Additive Explanations (SHAP) analysis. Model performance was evaluated in an independent test set using the area under the receiver operating characteristic curve (AUC), with sensitivity analyses performed using reduced feature sets.

**Results:**

Among 2,195 postoperative ICU patients, 694 (31.6%) developed CRAB infection. Patients with CRAB infection had significantly longer ICU stays, greater exposure to invasive procedures, higher antimicrobial use, and worse laboratory profiles than non-infected patients. Using 19 features selected by the Boruta algorithm, all eight machine learning models achieved good discrimination in the test set (AUC > 0.83). Gradient Boosting demonstrated the best overall performance, with an AUC of 0.867 (95% CI: 0.836–0.892), good calibration, and the highest net clinical benefit. SHAP analysis identified duration of mechanical ventilation, central venous catheterization, ICU length of stay (LOS), and carbapenem exposure as the most influential predictors. Sensitivity analyses showed that models using only the top 10 or top 5 SHAP-ranked features achieved performance comparable to the full model, supporting the feasibility of feature reduction for clinical application.

**Conclusions:**

This study provides an interpretable and clinically applicable framework for early risk assessment of CRAB infection in postoperative ICU patients, supporting targeted prevention strategies and more rational antimicrobial stewardship.

## Introduction

Carbapenem antibiotics have long been regarded as a critical “last line of defense” for the treatment of severe infections caused by multidrug-resistant Gram-negative bacteria (Papp-Wallace et al., 2011). With the widespread use of antimicrobial agents, carbapenem-resistant organisms (CROs) have continued to disseminate globally, among which carbapenem-resistant *Acinetobacter baumannii* (CRAB) has emerged as the most prominent pathogen (Yang et al., 2023). CRAB has been classified by the World Health Organization as a pathogen of “critical priority” on the list of antibiotic-resistant bacteria, representing a major challenge to infection prevention and control efforts worldwide (Tacconelli et al., 2018).

*Acinetobacter baumannii* is an important opportunistic pathogen capable of causing a wide spectrum of severe hospital-acquired infections, including ventilator-associated pneumonia, bloodstream infections, and wound infections (Russo and Serapide, 2025). Owing to its remarkable ability to adapt to adverse conditions, A. baumannii can persist and disseminate in the hospital environment for prolonged periods, with outbreaks occurring particularly frequently in intensive care units (ICUs) (Liu et al., 2022). ICU patients are at high risk for CRAB infection due to the severity of their underlying illnesses, compromised immune function, frequent exposure to invasive procedures, and extensive use of broad-spectrum antimicrobial agents (Medioli et al., 2025). Previous studies have demonstrated that CRAB infections among ICU patients are closely associated with significantly increased morbidity and mortality (Sharma and Lakhanpal, 2025). Consequently, postoperative ICU patients constitute an important and relatively distinct high-risk subgroup for CRAB infection. However, risk assessment tools specifically targeting this population remain limited.

Existing studies on CRAB have predominantly focused on describing epidemiological characteristics and identifying associated risk factors. Although multiple factors related to CRAB infection have been recognized, reliance on isolated or static risk factors in clinical practice is insufficient to achieve early and precise risk stratification at the individual patient level (Jiang et al., 2022). Some investigations have attempted to develop predictive models or scoring systems (Cogliati Dezza et al., 2023; He et al., 2024; Lan et al., 2024); however, most are based on traditional statistical approaches, which are limited in their ability to capture the nonlinear relationships and complex variable interactions that are common in ICU clinical data. Consequently, the generalizability and clinical applicability of these models remain suboptimal. Moreover, predictive models specifically tailored to postoperative ICU populations are particularly scarce.

In recent years, machine learning approaches have been increasingly applied to the development of clinical prediction models because of their advantages in handling high-dimensional, complex, and nonlinear data, and they have demonstrated considerable potential in infection risk prediction (Gao et al., 2024; Yang et al., 2025). However, conventional machine learning models are often regarded as “black boxes,” as their predictions lack interpretability, which limits clinicians’ understanding of and confidence in the model outputs and constrains their practical application in clinical decision-making (Guan et al., 2023). Explainable machine learning methods address this limitation by quantifying the contribution of individual predictive features to model outputs, thereby elucidating the underlying decision-making mechanisms and enhancing the transparency and interpretability of predictions. These methods can more effectively support clinical risk assessment and intervention strategies (Kim et al., 2024; Luo et al., 2025).

Given this background, we conducted a retrospective cohort study among patients admitted to the ICU after surgery. We systematically compared the performance of multiple machine learning models in predicting the risk of CRAB infection and integrated the Boruta feature selection algorithm with SHAP (Shapley Additive Explanations) interpretability analysis to develop and validate a prediction model with robust discriminative ability, good calibration, and strong clinical interpretability. This study aims to enable early identification of postoperative ICU patients at high risk for CRAB infection, to explore key driving factors and their interaction effects, and to provide evidence-based support for optimizing infection prevention and control strategies and promoting the rational use of antimicrobial agents in clinical practice.

## Methods

### Study design and population

**Figure 1:**
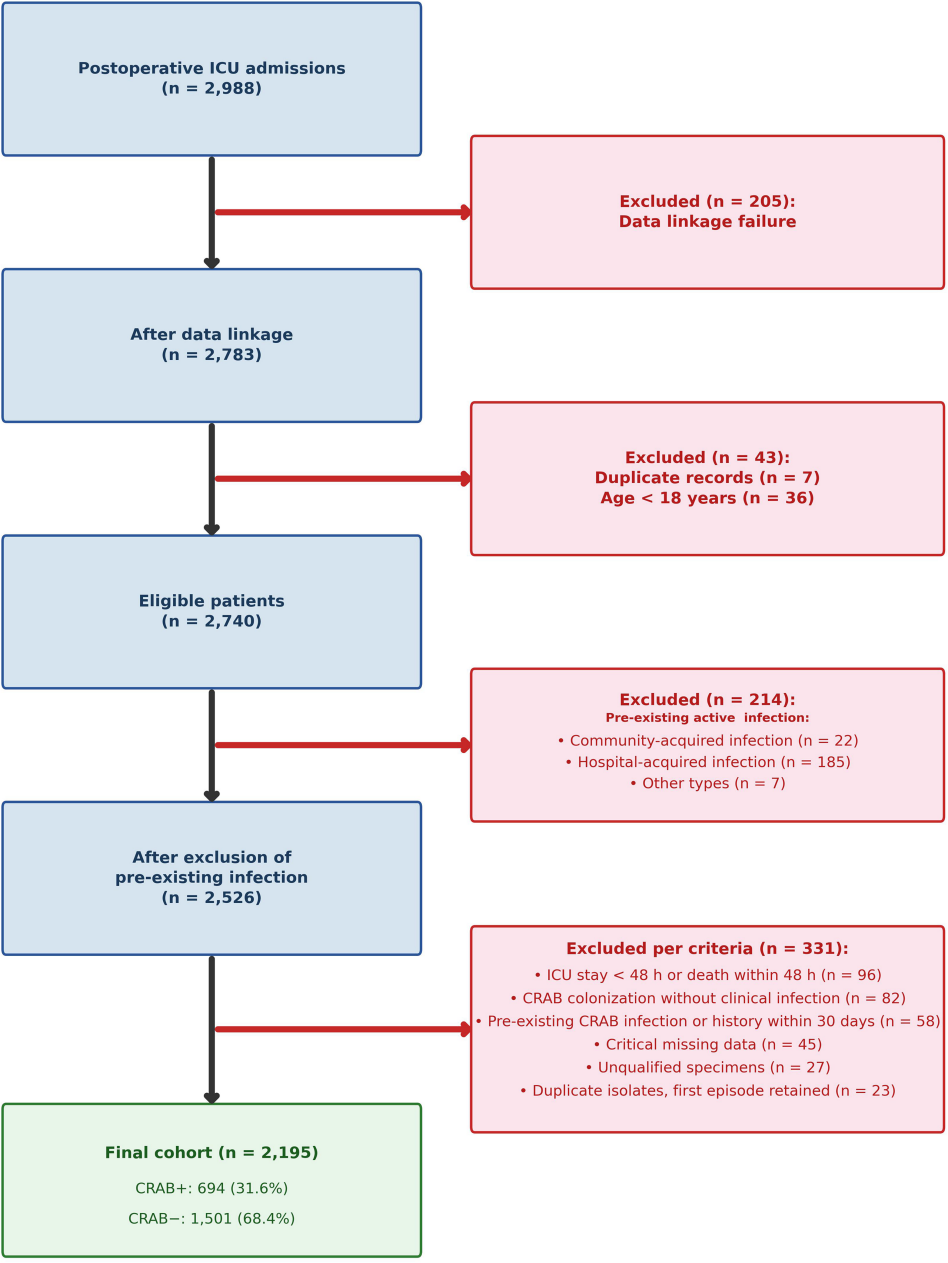
Participant flowchart.

This study was designed as a retrospective cohort study. Data were extracted from the electronic medical record system and the hospital infection surveillance database of a tertiary class A hospital in China. A total of 2,195 postoperative patients admitted to the ICU between January 1, 2023, and December 31, 2025, were included. Among them, 694 patients (31.6%) developed CRAB infection, while 1,501 patients (68.4%) did not. The participant selection process is illustrated in **Figure 1**.

Inclusion criteria:

1. Patients who underwent any surgical procedure during the study period and were admitted to the ICU postoperatively, aged ≥18 years.
2. Admission to the ICU within 24 hours after surgery, with an ICU length of stay (LOS) ≥48 hours.
3. During the ICU stay, qualified clinical specimens were submitted for microbiological testing, and *Acinetobacter baumannii* was isolated and identified by bacterial culture, with complete microbiological and antimicrobial susceptibility testing results available, allowing definitive determination of CRAB status.
4. The presence of clear infection-related clinical manifestations during the ICU stay, including but not limited to fever, purulent secretions, and signs and/or symptoms of organ- or system-specific infection.
5. The presence of one or more of the following supportive criteria for infection: elevated peripheral white blood cell (WBC) count, increased absolute neutrophil count or neutrophil percentage; elevated inflammatory biomarkers such as C-reactive protein (CRP) and/or procalcitonin (PCT); and/or imaging findings consistent with infection of the corresponding organ or system.
6. Marked improvement in clinical symptoms, physical signs, and laboratory parameters and/or imaging findings after 3–5 days of targeted or empirical antimicrobial therapy.
7. Complete records of infection-related clinical data, laboratory test results, and imaging findings during the ICU stay.

Exclusion criteria:

1. Patients with confirmed CRAB infection prior to ICU admission after surgery or within the first 48 hours after ICU admission.
2. Patients with a documented history of CRAB infection within 30 days before surgery that had not been fully resolved.
3. Patients with an ICU LOS <48 hours or who died within 48 hours after ICU admission.
4. Patients with isolation of AB alone but without any infection-related clinical manifestations, laboratory abnormalities, or imaging findings, who were therefore classified as colonization rather than infection.
5. Patients with unqualified clinical specimens or evident contamination, rendering the samples unsuitable for use as evidence for infection diagnosis.
6. Patients with incomplete clinical data, microbiological culture results, or antimicrobial susceptibility testing results, precluding definitive determination of CRAB infection.
7. For patients with multiple AB/CRAB isolates during the same hospitalization, only the first infection episode was included, and subsequent isolates were excluded.

### Pathogen identification and antimicrobial susceptibility testing

The first isolate of the target pathogen obtained from each patient was collected for analysis. Pathogens were isolated and cultured in accordance with the National Clinical Laboratory Operating Procedures (4th edition). Antimicrobial susceptibility testing was performed using the VITEK^®^ 2 Compact automated microbiology system (bioMérieux, France) with corresponding susceptibility testing cards. Susceptibility results were interpreted according to the Clinical and Laboratory Standards Institute (CLSI) M100 guidelines for antimicrobial susceptibility testing. Isolates of *Acinetobacter baumannii* with minimum inhibitory concentrations (MICs) ≥8 μg/mL for imipenem and/or meropenem were defined as CRAB.

### Bias control

Several potential sources of bias were identified and addressed. To minimize selection bias, rigorous inclusion and exclusion criteria were applied; the ICU stay ≥48 hours requirement aligned with the standard definition of healthcare-associated infections; patients with pre-existing active infections before ICU admission were excluded; and only the first infection episode per hospitalization was retained. To reduce detection bias, specimens collected at multiple time points during the ICU stay were included and all microbiological records were systematically reviewed. To address information bias, infection versus colonization was independently adjudicated by two investigators with third-party arbitration for disagreements, and data accuracy was ensured through cross-validation across the electronic medical record system, hospital infection surveillance database, and laboratory information system. During model development, feature selection procedures and the integration of multiple machine learning algorithms were employed to minimize the influence of confounding factors on predictive performance.

### Sample size

This study included all postoperative ICU patients who met the inclusion criteria between January 1, 2023, and December 31, 2025, representing a full-sample retrospective cohort. A total of 2,195 patients were enrolled, including 694 CRAB infection events (31.6%). The dataset was randomly split into a training set (70%, n = 1,537) and a validation set (30%, n = 658). Approximately 486 CRAB infection events were available in the training set with 19 candidate predictor variables, yielding an events per variable (EPV) ratio of approximately 25.6, which substantially exceeded the minimum recommended threshold of EPV ≥ 10 for the development of prediction models. According to the sample size framework for prediction models proposed by Riley et al., the sample size was considered adequate for stable model development and internal validation (Riley et al., 2020).

### Candidate predictor variables

Candidate predictive variables were determined based on clinical availability and evidence from previous studies and were primarily categorized as follows:

Demographic characteristics: age and sex.
ICU-related exposures, invasive procedures, and treatments: ICU LOS; exposure to central venous catheterization (CVC), mechanical ventilation (MV), and urinary catheterization (UC), as well as their respective durations (CVC days, MV days, UC days); surgery count; postoperative antibiotic days; total antibiotic days; and antibiotic combination therapy.
Exposure to antibiotic classes: cephalosporins, carbapenems, quinolones, glycopeptides, and aminoglycosides.
Underlying comorbidities: hypertension, diabetes, malignancy, renal dysfunction, cardiac disease, and hypoalbuminemia.
Laboratory parameters, including WBC (×10^9^/L), neutrophil pct (%), lymphocyte pct (%), CRP (mg/L), PCT (ng/mL), ALB (g/L), Hb (g/L), Cr (μmol/L), and PLT (×10^9^/L), were obtained from the first measurement within 48 hours after ICU admission.

### Data processing

Data processing was conducted in two stages. During the participant selection phase, 45 patients were excluded from the study cohort due to critical missing data in clinical records, microbiological culture results, or antimicrobial susceptibility testing results that precluded the definitive determination of CRAB infection status (**Figure 1**). Among the 2,195 patients included in the final cohort, missing data patterns were systematically assessed for all variables (**Supplementary Table 1**). The highest proportion of missing values was observed for PCT (n = 597, 27.20%), followed by ALB (n = 391, 17.81%), Cr (n = 315, 14.35%), CRP (n = 264, 12.03%), and surgery count (n = 235, 10.71%). WBC, neutrophil percentage, lymphocyte percentage, Hb, and PLT each had 33 missing values (1.50%). CVC days, MV days, UC days, and their corresponding binary variables each had 6 missing values (0.27%). All remaining variables had no missing data. As all missing proportions were below 30%, multiple imputation was performed to reduce potential bias (Pierce et al., 2023).

For continuous variables, outliers were detected using the interquartile range (IQR) method, whereby values below Q1 − 1.5 × IQR or above Q3 + 1.5 × IQR were flagged as potential outliers. Flagged values were reviewed against clinically plausible ranges: data entry errors were corrected, clinically implausible extreme values were handled by Winsorization (i.e., replaced with the corresponding boundary values of Q1 − 1.5 × IQR or Q3 + 1.5 × IQR), and extreme values judged by clinical experts to be genuine were retained to preserve clinically meaningful information.

### Feature selection

To reduce feature redundancy and mitigate the risk of overfitting, feature selection was conducted in two stages. First, Spearman correlation analysis was performed among all candidate predictors to assess multicollinearity. A threshold of |ρ| ≥ 0.9 was applied; when the absolute correlation coefficient between two variables reached or exceeded 0.9, the variable with the stronger association with the outcome was retained. The dataset was stratified and randomly split into training and testing sets at a ratio of 7:3, and feature selection was conducted exclusively within the training set to prevent information leakage. Subsequently, the Boruta algorithm, a wrapper-based feature selection method built upon the random forest (RF) classifier, was applied within the training set (Liu et al., 2025). For each original feature, a corresponding randomly permuted shadow feature was generated, and the importance Z-score distributions of real and shadow features were compared across repeated model training iterations. Features classified as “Confirmed” or “Tentative” under the statistical testing framework were retained as the final candidate input variables for subsequent model development and comparative evaluation (Rotari and Kulahci, 2024; Yan et al., 2023).

### Model development and hyperparameter optimization

Based on the candidate features identified above, eight machine learning classification models were developed: Gradient Boosting, RF, Light Gradient Boosting Machine (LightGBM), eXtreme Gradient Boosting (XGBoost), Extremely Randomized Trees (Extra Trees), Logistic Regression (LR), Support Vector Machine (SVM), and k-Nearest Neighbors (KNN).

Hyperparameter tuning was performed using Bayesian Optimization, with each model iterated 50 times within a predefined hyperparameter search space defined by the scikit-optimize library, using AUC-ROC as the optimization objective. Model stability and generalization were evaluated through 10-fold stratified cross-validation during training. After hyperparameters were determined, the optimal classification threshold for each model was identified by maximizing the Youden index on the training set. Final model performance was reported on the independent test set.

### Model evaluation and calibration

Discrimination performance was evaluated using the area under the receiver operating characteristic curve (AUC-ROC), along with sensitivity, specificity, accuracy, precision, negative predictive value, F1 score (Chicco and Jurman, 2020), and Matthews correlation coefficient (MCC). The 95% confidence intervals for all metrics were calculated using 1000 Bootstrap resampling iterations. Pairwise AUC comparisons between models were performed using the DeLong test, with P < 0.05 considered statistically significant.

Calibration performance was assessed using a multidimensional evaluation strategy. The Brier score was first calculated to quantify overall prediction accuracy. Calibration intercept and slope were then estimated by fitting a LR model on the logit-transformed predicted probabilities: a calibration intercept of 0 indicates the absence of systematic prediction bias, while a calibration slope of 1 indicates well-calibrated probability dispersion. The Hosmer-Lemeshow goodness-of-fit test was performed to evaluate whether predicted probabilities significantly deviated from observed event rates, with P > 0.05 indicating adequate calibration. For models with inadequate calibration (HL P ≤ 0.05), Platt Scaling with 5-fold cross-validation was applied for *post-hoc* recalibration, and all calibration metrics were reported both before and after recalibration.

Clinical decision utility was evaluated using Decision Curve Analysis (DCA). DCA quantifies the net benefit of each model across a range of threshold probabilities relative to the default strategies of “treat all” and “treat none,” and the integrated net benefit (INB) was calculated to summarize the overall decision advantage within the clinically relevant threshold range.

### Model interpretability analysis

To enhance clinical interpretability, the model with the best overall performance on the test set was selected, and the SHAP framework was applied to quantify feature contributions at both the individual and global levels. SHAP summary (importance) plots, force plots, and dependence plots were generated to illustrate the directional effects and cumulative impact of key variables on predicted probabilities, thereby providing interpretable evidence to support individualized risk assessment and prevention strategies for CRAB infection in postoperative ICU patients (Fan et al., 2023; Yi et al., 2023).

### Sensitivity analysis

To evaluate whether simplified feature sets achieved predictive performance comparable to the full model, the full feature set consisted of 19 variables selected using the Boruta algorithm, while reduced feature subsets including the top 10 and top 5 features were constructed based on SHAP importance ranking of the optimal model. Models were retrained for each feature set using the same pipeline as in the primary analysis. Model performance was assessed in the independent test set using the AUC, with 95% confidence intervals (CIs) estimated by bootstrap resampling. Differences in AUC between models with different feature sets were compared using the DeLong test, with P < 0.05 considered statistically significant.

### Statistical analysis

Continuous variables are presented as mean ± standard deviation (SD) or median (interquartile range, IQR), as appropriate, and categorical variables are expressed as numbers (percentages). Comparisons between continuous variables were performed using Student’s t test or the Mann–Whitney U test, as appropriate, while comparisons between categorical variables were conducted using the χ² test or Fisher’s exact test. All statistical tests were two sided, and a P value < 0.05 was considered statistically significant.

Statistical analyses and data visualization were performed using Python (version 3.9.13) and R (version 4.5.1).

## Results

### Comparison of baseline characteristics and candidate predictive variables

A total of 2,195 postoperative patients admitted to the ICU were included in this study, of whom 694 (31.6%) developed CRAB infection during their ICU stay. Baseline characteristics differed significantly between patients with and without CRAB infection with respect to ICU exposure, invasive procedures, antimicrobial use, and multiple clinical parameters. Patients with CRAB infection had significantly more ICU days than those without infection (19 vs. 9 days, P < 0.001). CVC days, MV days, and UC days were also significantly higher in the CRAB infection group (all P < 0.001). The median surgery count was higher in patients with CRAB infection (P < 0.001) (**Table 1**).

**Table 1:** Baseline characteristics of postoperative ICU patients stratified by CRAB infection status.

Variable	Total (n=2195)	CRAB- (n=1501)	CRAB+ (n=694)	P-value
Age	63 (53-70)	63 (53-69)	62 (52-71)	0.661
LOS	11 (7-20)	9 (6-14)	19 (12-29)	<0.001
CVC days	7 (0-20)	3 (0-12)	20 (10-31)	<0.001
MV days	6 (1-14)	4 (0-9)	14 (8-24)	<0.001
UC days	13 (6-22)	11 (6-18)	18 (10-29)	<0.001
Surgery count	2 (1-3)	2 (1-3)	2 (1-3)	<0.001
Postoperative antibiotic days	2 (2-10)	2 (2-10)	2 (2-10)	0.929
Antibiotics days	19 (12-29)	16 (11-24)	26 (18-38)	<0.001
Antibiotics combination	1 (1-1)	1 (1-1)	1 (1-1)	<0.001
WBC, ×10^9^/L	11.30 (8.50-14.60)	11.30 (8.40-14.40)	11.30 (8.72-14.90)	0.448
Neutrophil pct, %	85.70 (79.90-89.70)	86.00 (80.30-89.90)	85.00 (79.30-89.30)	0.043
Lymphocyte pct, %	8.10 (5.30-12.30)	7.90 (5.30-12.10)	8.40 (5.33-12.60)	0.282
CRP, mg/L	61.25 (19.71-112.96)	58.45 (16.26-108.15)	67.57 (27.56-120.30)	<0.001
PCT, ng/mL	0.82 (0.20-3.21)	0.67 (0.17-2.55)	1.32 (0.25-5.22)	<0.001
ALB, g/L	31.90 (29.00-35.90)	32.30 (29.40-36.80)	31.00 (28.23-34.50)	<0.001
Hb, g/L	114 (95-133)	118 (99-135)	106 (88-127)	<0.001
Cr, μmol/L	77.60 (57.78-111.04)	75.20 (57.20-104.42)	82.57 (60.00-131.68)	<0.001
PLT, ×10^9^/L	184 (129-246)	186 (136-243)	179 (115-253)	0.194
Sex				0.075
Male	1546 (70.4%)	1039 (69.2%)	507 (73.1%)	
Female	649 (29.6%)	462 (30.8%)	187 (26.9%)	
CVC				<0.001
No	762 (34.7%)	684 (45.6%)	78 (11.2%)	
Yes	1433 (65.3%)	817 (54.4%)	616 (88.8%)	
MV				<0.001
No	467 (21.3%)	426 (28.4%)	41 (5.9%)	
Yes	1728 (78.7%)	1075 (71.6%)	653 (94.1%)	
UC				0.047
No	94 (4.3%)	55 (3.7%)	39 (5.6%)	
Yes	2101 (95.7%)	1446 (96.3%)	655 (94.4%)	
Cephalosporins				<0.001
No	1770 (80.6%)	1240 (82.6%)	530 (76.4%)	
Yes	425 (19.4%)	261 (17.4%)	164 (23.6%)	
Carbapenems				<0.001
No	1848 (84.2%)	1368 (91.1%)	480 (69.2%)	
Yes	347 (15.8%)	133 (8.9%)	214 (30.8%)	
Quinolones				0.056
No	2149 (97.9%)	1476 (98.3%)	673 (97.0%)	
Yes	46 (2.1%)	25 (1.7%)	21 (3.0%)	
Glycopeptides				<0.001
No	2136 (97.3%)	1474 (98.2%)	662 (95.4%)	
Yes	59 (2.7%)	27 (1.8%)	32 (4.6%)	
Aminoglycosides				1.000
No	2150 (97.9%)	1470 (97.9%)	680 (98.0%)	
Yes	45 (2.1%)	31 (2.1%)	14 (2.0%)	
Hypertension				<0.001
No	1348 (61.4%)	864 (57.6%)	484 (69.7%)	
Yes	847 (38.6%)	637 (42.4%)	210 (30.3%)	
Diabetes				0.171
No	1859 (84.7%)	1260 (83.9%)	599 (86.3%)	
Yes	336 (15.3%)	241 (16.1%)	95 (13.7%)	
Malignancy				0.132
No	2096 (95.5%)	1426 (95.0%)	670 (96.5%)	
Yes	99 (4.5%)	75 (5.0%)	24 (3.5%)	
Renal dysfunction				<0.001
No	1838 (83.7%)	1314 (87.5%)	524 (75.5%)	
Yes	357 (16.3%)	187 (12.5%)	170 (24.5%)	
Cardiac disease				0.018
No	1792 (81.6%)	1205 (80.3%)	587 (84.6%)	
Yes	403 (18.4%)	296 (19.7%)	107 (15.4%)	
Hypoalbuminemia				<0.001
No	1661 (75.7%)	1200 (79.9%)	461 (66.4%)	
Yes	534 (24.3%)	301 (20.1%)	233 (33.6%)	

With respect to antimicrobial-related factors, postoperative antibiotic days and antibiotics days were both significantly higher in the CRAB infection group (26 vs. 16 days, P < 0.001), and the proportion of patients receiving antibiotics combination therapy was higher (P < 0.001). Prior exposure to carbapenems and glycopeptides was more frequent in patients with CRAB infection (P < 0.001).Regarding comorbidities and laboratory parameters, the prevalences of renal dysfunction, cardiac disease, and hypoalbuminemia were significantly higher in the CRAB infection group than in the non-infection group (all P < 0.001). Laboratory results showed higher Neutrophil pct and lower Lymphocyte pct, along with lower Hb and ALB levels and higher Cr levels in patients with CRAB infection; all differences were statistically significant (all P < 0.001) (**Table 1**).

### Feature selection

**Figure 2:**
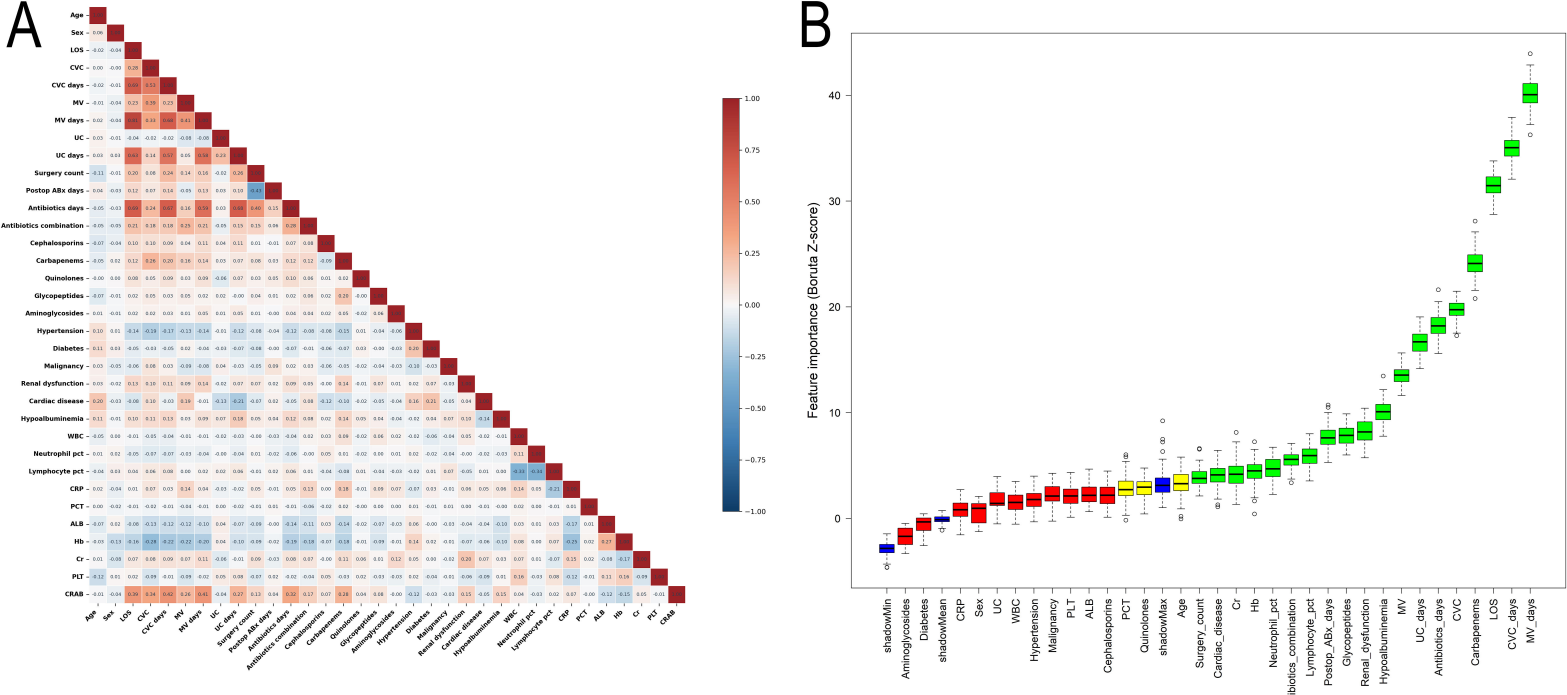
Feature selection process. **(A)** Correlation heatmap of candidate variables. **(B)** Feature importance ranking using Boruta algorithm.

Prior to model development, correlation analysis was performed for all candidate predictors to assess the risk of multicollinearity. As shown in the correlation heatmap (**Figure 2A**), the absolute values of Spearman correlation coefficients between all pairs of continuous and categorical variables did not exceed 0.9, indicating the absence of strong correlations. Therefore, all candidate variables were retained for subsequent feature selection.

Feature selection was then conducted using the RF–based Boruta algorithm. A total of 19 variables were identified and included in subsequent model construction (**Figure 2B**). The selected variables were mainly distributed across categories related to ICU exposure and invasive procedures, antimicrobial use, comorbidities, and laboratory parameters. Variables related to ICU exposure and invasive procedures included LOS, CVC, CVC days, MV, MV days, and UC days. Treatment-related variables comprised surgery count, postoperative antibiotic days, antibiotics days, and antibiotics combination. Among antimicrobial-related variables, carbapenems and glycopeptides were defined as exposure within 7 days prior to ICU admission. Comorbidity variables included renal dysfunction, cardiac disease, and hypoalbuminemia. Laboratory parameters included Neutrophil pct, Lymphocyte pct, Hb, and Cr.

### Development and evaluation of prediction models

**Figure 3:**
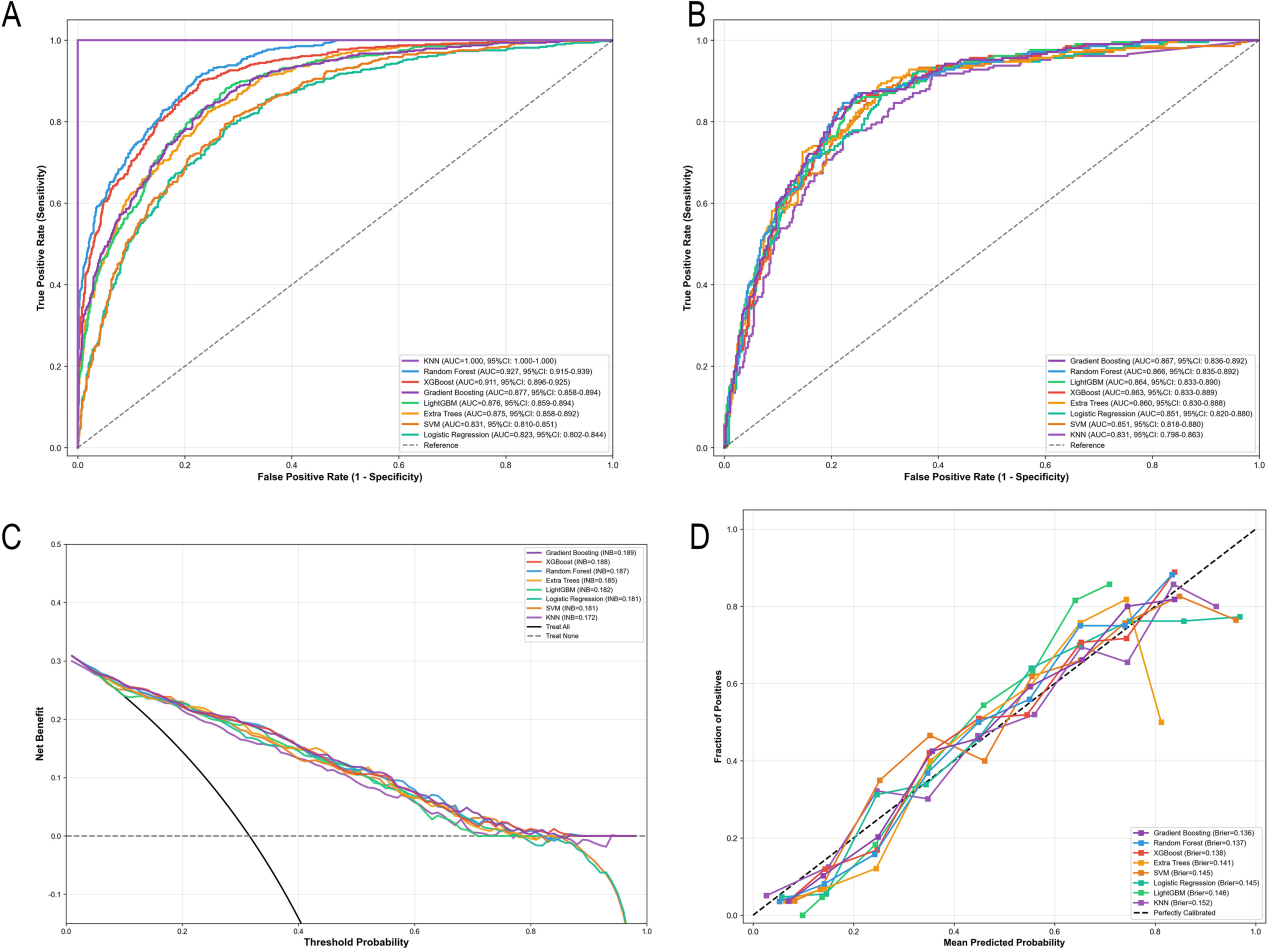
Performance evaluation of machine learning models. **(A)** ROC curves on the training set. **(B)** ROC curves on the test set. **(C)** Decision curve analysis on the test set. **(D)** Calibration curves on the test set.

Based on the 19 features selected using the Boruta algorithm, eight machine learning models were developed to predict the risk of CRAB infection during the postoperative ICU stay. ROC curve analysis in the test set demonstrated that all models achieved AUC values greater than 0.83. Among them, Gradient Boosting showed the best discrimination performance (AUC = 0.867, 95% CI: 0.836–0.892), followed closely by RF (AUC = 0.866) and LightGBM (AUC = 0.864). LR and SVM yielded identical AUC values of 0.851, whereas KNN showed the lowest performance (AUC = 0.831) (**Figures 3A, B**; **Table 2**).

**Table 2:** Performance comparison of machine learning models on the test set.

Model	AUC	Sensitivity	Specificity	Precision	F1	MCC	Accuracy
Gradient Boosting	0.867 (0.836-0.892)	0.841 (0.790-0.887)	0.763 (0.722-0.800)	0.622 (0.567-0.674)	0.715 (0.670-0.755)	0.568 (0.506-0.627)	0.788 (0.756-0.818)
Random Forest	0.866 (0.835-0.892)	0.788 (0.727-0.842)	0.805 (0.768-0.841)	0.652 (0.591-0.709)	0.713 (0.667-0.757)	0.568 (0.498-0.630)	0.800 (0.766-0.830)
LightGBM	0.864 (0.833-0.890)	0.850 (0.799-0.896)	0.752 (0.712-0.792)	0.614 (0.557-0.668)	0.713 (0.668-0.753)	0.565 (0.504-0.624)	0.783 (0.751-0.815)
XGBoost	0.863 (0.833-0.889)	0.831 (0.779-0.881)	0.785 (0.746-0.824)	0.642 (0.586-0.696)	0.724 (0.676-0.766)	0.583 (0.519-0.643)	0.800 (0.768-0.830)
Extra Trees	0.860 (0.830-0.888)	0.827 (0.773-0.878)	0.746 (0.704-0.784)	0.601 (0.545-0.656)	0.696 (0.650-0.738)	0.537 (0.472-0.597)	0.771 (0.737-0.801)
Logistic Regression	0.851 (0.820-0.880)	0.823 (0.768-0.875)	0.728 (0.686-0.765)	0.584 (0.529-0.634)	0.683 (0.635-0.727)	0.516 (0.448-0.577)	0.758 (0.724-0.791)
SVM	0.851 (0.818-0.880)	0.871 (0.822-0.918)	0.715 (0.671-0.756)	0.586 (0.531-0.637)	0.700 (0.653-0.744)	0.546 (0.480-0.603)	0.764 (0.730-0.795)
KNN	0.831 (0.798-0.863)	0.952 (0.921-0.979)	0.416 (0.372-0.458)	0.430 (0.386-0.475)	0.592 (0.548-0.635)	0.374 (0.323-0.420)	0.586 (0.549-0.621)

Comparisons between the training and test sets revealed evident overfitting in the KNN model, with an AUC of 1.000 in the training set and 0.831 in the test set. In contrast, Gradient Boosting demonstrated good generalization ability, with comparable AUC values in the training (0.877) and test (0.867) sets. After determining the optimal cutoff values using the Youden index, model sensitivities ranged from 0.788 to 0.952, while specificities ranged from 0.416 to 0.805. XGBoost achieved the highest F1 score (0.724), with a sensitivity of 0.831 and a specificity of 0.785. RF yielded the highest specificity (0.805) with a sensitivity of 0.788. LightGBM showed relatively higher sensitivity (0.850) with a specificity of 0.752. Gradient Boosting achieved a sensitivity of 0.841, a specificity of 0.763, and an F1 score of 0.715, indicating a well-balanced overall performance (**Table 2**).

**Figure 4:**
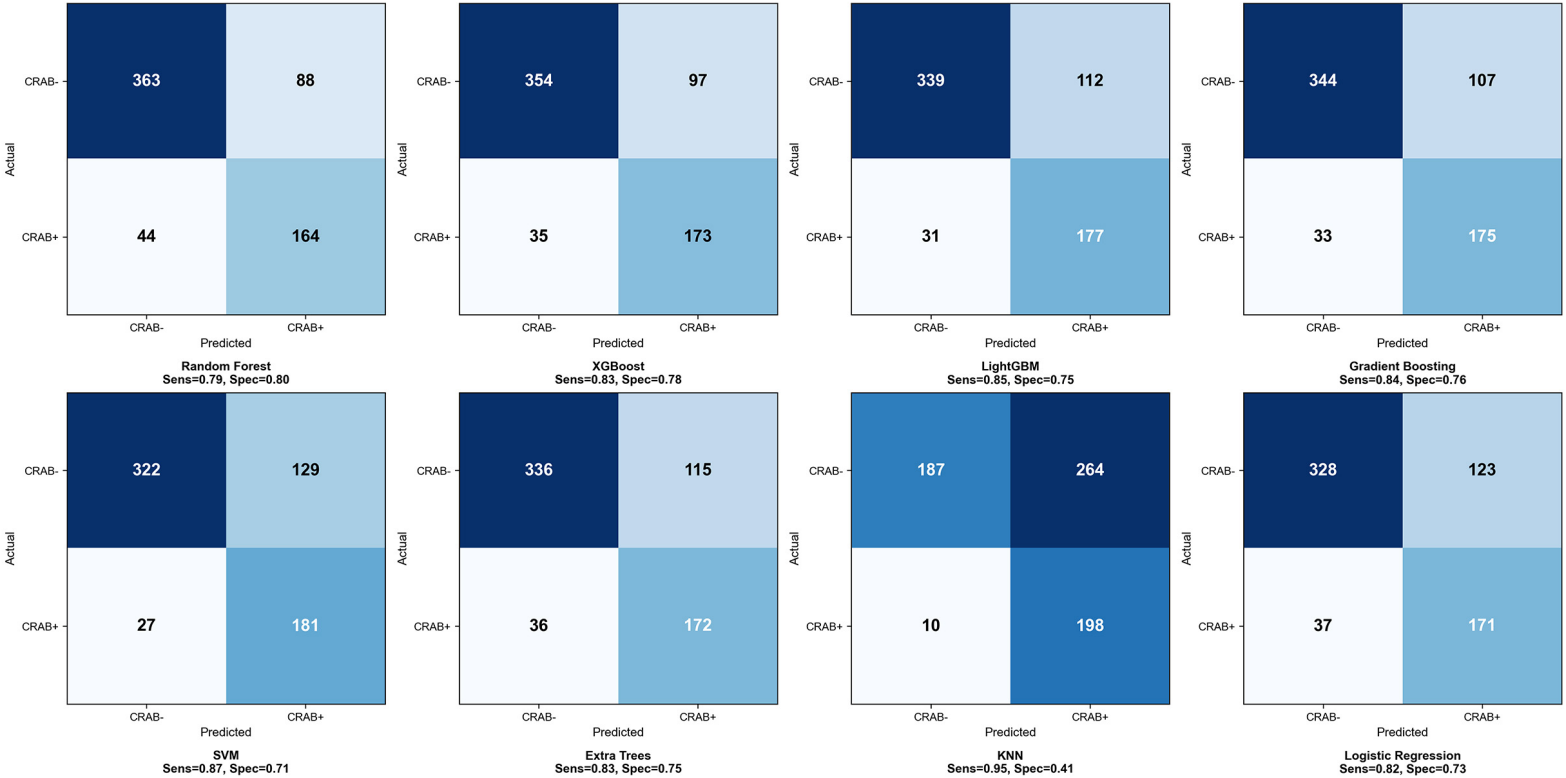
Confusion matrices of eight machine learning models on the test set.

The confusion matrix indicated that Gradient Boosting correctly identified 175 CRAB-positive and 344 CRAB-negative cases, with 33 false negatives and 107 false positives. RF produced the fewest false positives (88 cases) but more false negatives (44 cases) (**Figure 4**). Regarding calibration performance, pre- and post-calibration metrics for all eight models are detailed in **Supplementary Table 3**. In the pre-calibration assessment, Gradient Boosting, XGBoost, and SVM demonstrated adequate calibration, with Hosmer-Lemeshow test P-values of 0.193, 0.090, and 0.107, respectively. The remaining five models showed inadequate calibration (P ≤ 0.05) and underwent Platt Scaling recalibration. After recalibration, RF (P = 0.086) and KNN (P = 0.095) achieved adequate calibration, whereas LightGBM (P = 0.016), Extra Trees (P = 0.027), and LR (P = 0.014) remained inadequately calibrated. Notably, XGBoost exhibited a decline in calibration after Platt Scaling (HL P-value decreased from 0.090 to 0.017), suggesting an over-calibration effect; therefore, the pre-calibration version was retained. Overall, Gradient Boosting demonstrated the best calibration performance, with a calibration intercept (0.036) and slope (1.218) closest to ideal values and the lowest Brier score (0.136) (**Supplementary Table 3**; **Figure 3D**). In DCA, within a threshold probability range of 0.1–0.5, all models yielded a higher net benefit than the “treat-all” and “treat-none” strategies. Gradient Boosting achieved the highest integrated net benefit (INB = 0.189) (**Figure 3C**). Considering discrimination ability, generalization performance, calibration, and clinical decision value, the Gradient Boosting model demonstrated consistently strong and well-balanced performance across all dimensions and was therefore selected as the optimal predictive model in this study.

### Model interpretability analysis based on SHAP

**Figure 5:**
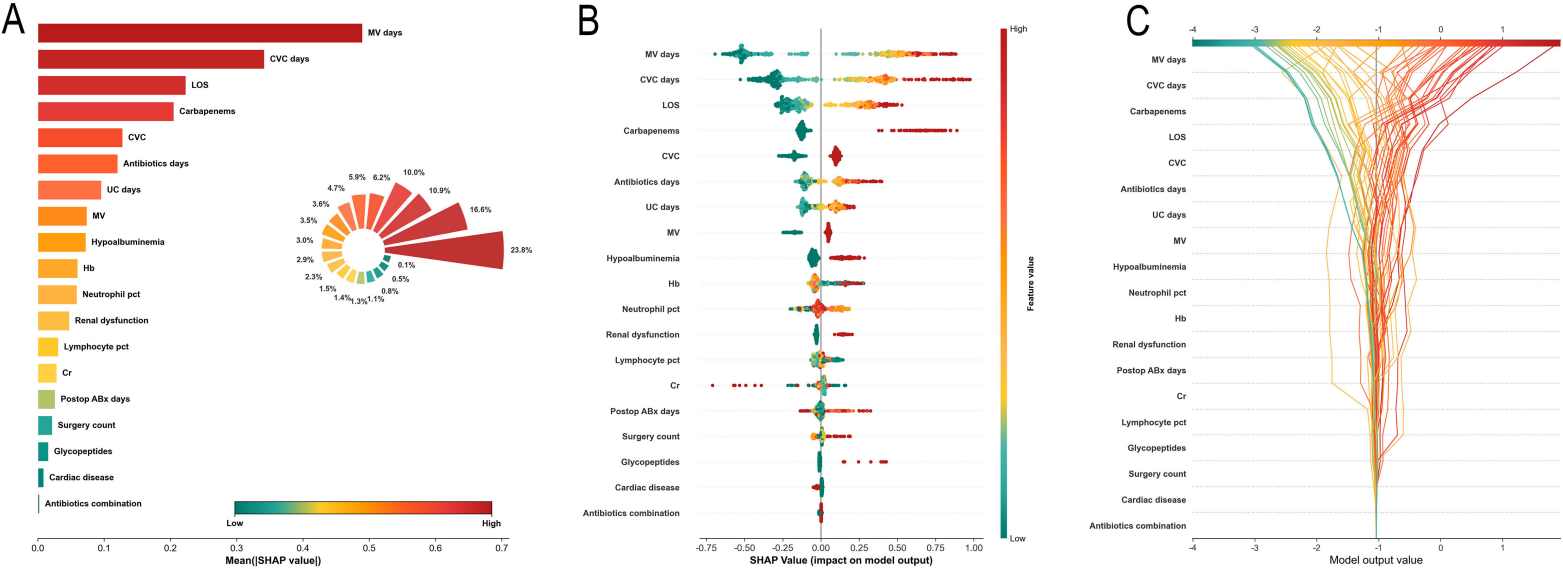
SHAP interpretation of the Gradient Boosting model. **(A)** Feature importance ranking with bar chart and rose plot showing percentage contributions. **(B)** SHAP beeswarm plot displaying feature effects on model output. **(C)** SHAP decision plot illustrating cumulative feature contributions for individual predictions.

SHAP analysis was performed to interpret the Gradient Boosting model. Feature importance ranking indicated that MV days contributed the most to model predictions (23.8%), followed by CVC days (16.6%), LOS (10.9%), and Carbapenems use (10.0%); together, the top four features accounted for more than 60% of the total contribution (**Figure 5A**).

The SHAP beeswarm plot illustrated the directional associations between individual features and the risk of CRAB infection. Continuous variables, including MV days, CVC days, LOS, antibiotics days, and UC days, showed clear positive effects, with higher values corresponding to larger SHAP values and increased infection risk (**Figure 5B**). For binary variables, positive status—including Carbapenems use, Glycopeptides use, MV, CVC, hypoalbuminemia, and renal dysfunction—was consistently associated with an increased risk of CRAB infection. SHAP dependence plots further demonstrated monotonic, nonlinear increases in SHAP values with increasing MV days, CVC days, and LOS. In contrast, Neutrophil pct exhibited a dispersed SHAP value distribution at higher levels, suggesting interindividual heterogeneity in its impact on CRAB infection risk (**Supplementary Figure 2**).

The SHAP decision plot illustrated the cumulative trajectories of test-set samples from the baseline value to the final prediction. High-risk samples showed pronounced rightward shifts at the MV days and CVC days levels, whereas low-risk samples exhibited a sustained leftward trajectory (**Figure 5C**). The SHAP waterfall plots demonstrated feature contributions to individual predictions using three representative cases. Low-risk cases were primarily driven by shorter durations of invasive procedures, whereas high-risk cases were jointly influenced by CVC days, MV days, Carbapenems use, and LOS (**Supplementary Figure 1**). SHAP interaction dependence plots revealed positive interaction effects between LOS and MV days, CVC days, and Carbapenems use, indicating that prolonged ICU stay combined with extended invasive procedures or carbapenem exposure further amplified the risk of CRAB infection (**Supplementary Figure 3**).

### Sensitivity analysis for feature reduction

Sensitivity analysis demonstrated no significant differences in predictive performance between the reduced-feature models and the full model. In the Top-10 feature model, Gradient Boosting achieved an AUC of 0.869 (95% CI: 0.840–0.894), which did not differ significantly from that of the full model (AUC = 0.867; P = 0.522). In the Top-5 feature model, the AUC of Gradient Boosting was 0.867 (95% CI: 0.836–0.892), virtually identical to the full model (P = 0.975) (**Supplementary Table 2**; **Supplementary Figure 4**).

For the other seven models, performance on both reduced feature subsets showed no significant differences compared with the full model, with all DeLong test P values > 0.05 and AUC changes ranging from −0.018 to +0.010. Notably, RF exhibited the largest AUC decrease in the Top-5 feature model (0.866 to 0.856); however, this reduction did not reach statistical significance (P = 0.087) (**Supplementary Table 4**). These findings indicate that a simplified model incorporating only the top five SHAP-ranked features (MV days, CVC days, LOS, Carbapenems use, and CVC) can achieve predictive performance comparable to that of the full model, providing a practical basis for streamlined clinical implementation.

## Discussion

In this study, a predictive model for CRAB infection risk was developed based on a cohort of 2,195 postoperative patients admitted to the ICU. Nineteen candidate predictors were identified using the Boruta algorithm, and the predictive performance of eight machine learning algorithms was systematically compared. Gradient Boosting was ultimately selected as the optimal model, achieving an AUC of 0.867 (95% CI: 0.836–0.892). This model demonstrated not only strong discrimination ability but also favorable calibration performance, yielding the highest net benefit in DCA, thereby supporting its potential clinical utility. SHAP-based interpretability analysis identified MV days, CVC days, LOS, and Carbapenems use as the most influential risk factors for CRAB infection, with the top four features accounting for more than 60% of the total contribution. Sensitivity analysis further confirmed that a simplified model incorporating only the top five SHAP-ranked features achieved predictive performance comparable to that of the full model, providing a rationale for streamlined implementation in clinical practice.

In this study, the incidence of CRAB infection was 31.6% (694/2,195), representing a moderate-to-high level when compared with global epidemiological data. A European meta-analysis reported a weighted mean prevalence of carbapenem-non-susceptible *Acinetobacter baumannii* of 35.6% (95% CI: 29.7%–42.0%), with the proportion among ICU isolates reaching 54.0% (95% CI: 47.6%–60.3%) (Ayobami et al., 2020); the burden was particularly severe in Southern and Eastern Europe (75.5% and 71.5%, respectively), whereas substantially lower rates were observed in Northern and Western Europe (2.8% and 6.3%) (Ayobami et al., 2020). Data from 70 healthcare facilities in the United States indicated that 29.9% of ICUs reported cases of carbapenem-non-susceptible A. baumannii (Sader et al., 2022). In China, a previous study reported that the proportion of CRAB among ICU-acquired A. baumannii infections was as high as 76.5%, markedly exceeding that reported in Europe and the United States (Li et al., 2025). The present study focused on a specific population of postoperative patients admitted to the ICU. Although the observed CRAB infection rate (31.6%) was lower than the overall CRAB detection rates reported in Chinese ICU settings, it nonetheless reflects the substantial burden of antimicrobial-resistant infections faced by postoperative ICU patients in China. Since its first identification in 1985, CRAB has disseminated globally and has been designated by the World Health Organization as a “critical priority” pathogen. The mortality associated with CRAB infection exceeds 20%, with bloodstream infection–related mortality surpassing 40%, accounting for more than 50,000 deaths worldwide each year (Müller et al., 2023). Given the pronounced environmental resilience of CRAB, its ability to form biofilms, and the limited therapeutic options currently available, early identification of high-risk patients is of considerable clinical importance for the timely implementation of infection prevention and control measures.

MV days, Carbapenems use, and CVC days were identified as the most influential predictors in our model, consistent with findings from previous studies (Qiao et al., 2020; Zhang et al., 2023; Zhou et al., 2014). A CRAB-focused study reported that mechanical ventilation was an independent risk factor for CRAB infection (OR = 3.2, 95% CI: 1.8–5.6) (Li et al., 2025). Invasive procedures may compromise the integrity of the skin barrier, facilitating colonization of CRAB strains on the skin and subsequent invasion into deeper tissues. In addition, CRAB possesses an intrinsic ability to form biofilms on indwelling devices, enabling migration to epithelial cells of underlying tissues and ultimately leading to infection (Yang et al., 2019).

Prior exposure to carbapenems, identified as the fourth most important predictor (10.0%), likely exerts its effect through antibiotic selection pressure. Previous studies have demonstrated that carbapenem exposure for ≥48 hours is an independent risk factor for CRAB infection (OR = 1.89, 95% CI: 1.32–2.71) (Li et al., 2025). Carbapenem resistance in CRAB is primarily mediated by plasmid-borne carbapenemase genes, particularly blaOXA-23 (Kyriakidis et al., 2021). Additional mechanisms include loss or mutation of the outer membrane protein CarO, which reduces outer membrane permeability (Catel-Ferreira et al., 2011; Poirel and Nordmann, 2006; Siroy et al., 2005), and the involvement of AbuO in active efflux of carbapenems (Srinivasan et al., 2015; Vila et al., 2007). The use of broad-spectrum antibiotics provides a selective environment that facilitates the emergence and enrichment of these resistance mechanisms. Notably, SHAP beeswarm plots in the present study also indicated that glycopeptides use was associated with an increased risk of infection, underscoring the importance of antimicrobial stewardship and avoidance of unnecessary broad-spectrum antibiotic exposure in clinical practice.

LOS (10.9%), ranked as the third most important feature, has likewise been consistently associated with CRAB infection risk. Prior research has shown that hospitalization exceeding 14 days significantly increases the risk of CRAB infection (OR = 1.67, 95% CI: 1.25–2.23) (Li et al., 2025). Furthermore, SHAP interaction analysis in this study revealed positive interaction effects between LOS and MV days as well as CVC days, suggesting that prolonged hospitalization combined with extended exposure to invasive procedures synergistically amplifies infection risk. These findings provide evidence-based support for timely assessment of weaning and device removal and for minimizing unnecessary durations of invasive interventions in ICU settings.

Gradient Boosting was selected as the optimal model rather than RF or XGBoost, despite their comparable AUC values, based on an integrated assessment of overall performance. First, Gradient Boosting demonstrated the most stable generalization ability, with a minimal AUC difference of only 0.01 between the training and test sets (0.877 vs. 0.867). Second, Gradient Boosting exhibited the best calibration performance (Brier score = 0.136), outperforming RF (0.137) and LightGBM (0.146). This indicates that its predicted probabilities were more closely aligned with observed risks, which is critical for determining intervention thresholds in clinical decision-making. Poorly calibrated models, even with strong discrimination, may lead to overestimation or underestimation of risk and consequently compromise clinical decision accuracy. Third, DCA showed that Gradient Boosting achieved the highest integrated net benefit within a threshold probability range of 0.1–0.5 (INB = 0.189), suggesting that it would confer the greatest net clinical benefit when applied in practice.

Compared with previously reported models, the present model demonstrates favorable predictive performance. Existing prediction models for carbapenem-resistant infections in ICU settings generally report AUC values ranging from 0.72 to 0.87 and predominantly rely on traditional LR, with variable selection largely based on univariable analyses and expert opinion [14.1]. In contrast, this study employed the Boruta algorithm for feature selection, thereby reducing subjective bias. Bayesian optimization was used for hyperparameter tuning to fully exploit model capacity, while SHAP analysis provided not only global feature importance rankings but also insights into feature interactions and individualized prediction explanations.

Sensitivity analysis further demonstrated that a parsimonious model incorporating only the top five features (MV days, CVC days, LOS, Carbapenems use, and CVC) achieved predictive performance comparable to that of the full model (AUC = 0.867, P = 0.975). This finding supports the feasibility of developing a simplified clinical risk scoring tool, which may reduce data collection burden and enhance the accessibility and applicability of the model in real-world clinical settings.

This study has several limitations. First, as a single-center retrospective study, the external generalizability of the model requires further validation, as CRAB epidemiology and resistance patterns may vary across regions and healthcare settings. Second, the study population was limited to postoperative ICU patients, and the applicability of the model to non-surgical ICU patients or general ward populations remains uncertain. Third, pathogen-related characteristics and clinical outcomes were not included, and the temporal relationships of certain time-varying variables could not be fully clarified, raising the possibility of reverse causality. Finally, the clinical implementation and real-world impact of the model were not evaluated and warrant further investigation.

## Conclusion

This study established a clinically applicable model for predicting the risk of CRAB infection among postoperative ICU patients. The model demonstrated reliable predictive performance and identified key risk factors primarily related to invasive procedures, antibiotic exposure, and ICU LOS. A simplified version using a limited number of core predictors achieved performance comparable to the full model, supporting its potential for practical clinical use. Early risk stratification using this approach may facilitate timely infection prevention and control interventions in postoperative ICU settings.

## Data Availability

The original contributions presented in the study are included in the article/**Supplementary Material**. Further inquiries can be directed to the corresponding author.
