# Supplementary material for: Development and validation of an interpretable machine learning–based model for predicting carbapenem-resistant Acinetobacter baumannii infection in postoperative ICU patients: a retrospective cohort study
Source: Front Cell Infect Microbiol. 2026 Mar 25;16:1788229. doi: 10.3389/fcimb.2026.1788229 (PMC13057271; doi:10.3389/fcimb.2026.1788229)
Supplement: Supplementary file 1 [file Table1.docx]

**Table S1 Number and proportion of missing values for each variable before multiple imputation (N = 2195)**

| **variable** | **Count of missing values** | **Proportion of missing values** |
| --- | --- | --- |
| age | 0 | 0 |
| sex | 0 | 0 |
| ICU LOS | 0 | 0 |
| CVC days | 6 | 0.002733485 |
| MV days | 6 | 0.002733485 |
| UC days | 6 | 0.002733485 |
| surgery count | 235 | 0.107061503 |
| postoperative antibiotic days | 0 | 0 |
| total antibiotic days | 0 | 0 |
| antibiotic combination therapy | 0 | 0 |
| central venous catheterization (CVC) | 6 | 0.002733485 |
| mechanical ventilation (MV) | 6 | 0.002733485 |
| urinary catheterization (UC) | 6 | 0.002733485 |
| cephalosporins | 0 | 0 |
| carbapenems | 0 | 0 |
| quinolones | 0 | 0 |
| glycopeptides | 0 | 0 |
| aminoglycosides | 0 | 0 |
| hypertension | 0 | 0 |
| diabetes | 0 | 0 |
| malignancy | 0 | 0 |
| renal dysfunction | 0 | 0 |
| cardiac disease | 0 | 0 |
| hypoalbuminemia | 0 | 0 |
| WBC (×10⁹/L) | 33 | 0.015034169 |
| neutrophil pct (%) | 33 | 0.015034169 |
| lymphocyte pct (%) | 33 | 0.015034169 |
| CRP (mg/L) | 264 | 0.120273349 |
| PCT (ng/mL) | 597 | 0.271981777 |
| ALB (g/L) | 391 | 0.178132118 |
| Hb (g/L) | 33 | 0.015034169 |
| Cr (μmol/L) | 315 | 0.143507973 |
| PLT (×10⁹/L) | 33 | 0.015034169 |

**Table S2. Sensitivity analysis: AUC values (95% CI) of eight machine learning models using all features (n=19), top 10 features, and top 5 features. Delta values indicate AUC change relative to the full model.**

| **Model** | **All_AUC** | **Top10_AUC** | **Top5_AUC** | **Delta_Top10** | **Delta_Top5** |
| --- | --- | --- | --- | --- | --- |
| XGBoost | 0.863 (0.833-0.889) | 0.867 (0.838-0.891) | 0.866 (0.835-0.891) | -0.0036 | -0.0028 |
| Random Forest | 0.866 (0.835-0.892) | 0.867 (0.836-0.892) | 0.856 (0.821-0.884) | -0.0005 | 0.0102 |
| LightGBM | 0.864 (0.833-0.890) | 0.868 (0.839-0.892) | 0.864 (0.833-0.890) | -0.0039 | 0.0001 |
| Gradient Boosting | 0.867 (0.836-0.892) | 0.869 (0.840-0.894) | 0.867 (0.836-0.892) | -0.0019 | 0.0002 |
| SVM | 0.851 (0.818-0.880) | 0.850 (0.819-0.878) | 0.854 (0.822-0.882) | 0.0009 | -0.0036 |
| Extra Trees | 0.860 (0.830-0.888) | 0.861 (0.831-0.886) | 0.857 (0.824-0.885) | -0.0006 | 0.0032 |
| KNN | 0.831 (0.798-0.863) | 0.838 (0.806-0.868) | 0.849 (0.813-0.881) | -0.0082 | -0.0185 |
| Logistic Regression | 0.851 (0.820-0.880) | 0.857 (0.827-0.884) | 0.856 (0.824-0.884) | -0.0054 | -0.0048 |

**Table S3. Calibration performance of machine learning models before and after Platt Scaling recalibration.**

| **Model** | **Pre-calibration** | | | | **Post-calibration (Platt Scaling)** | | | |
| --- | --- | --- | --- | --- | --- | --- | --- | --- |
|  | **Brier Score** | **Intercept** | **Slope** | **HL P-value** | **Brier Score** | **Intercept** | **Slope** | **HL P-value** |
| Gradient Boosting | 0.1364 | 0.0355 | 1.2176 | 0.1931 | 0.1358 | 0.0604 | 1.2602 | 0.1392 |
| XGBoost | 0.138 | 0.0336 | 1.2239 | 0.0897 | 0.139 | 0.0636 | 1.2796 | 0.017 |
| Random Forest | 0.1374 | 0.096 | 1.3026 | 0.0229 | 0.137 | 0.0936 | 1.2823 | 0.086 |
| LightGBM | 0.1456 | 0.3913 | 1.7663 | <0.001 | 0.138 | 0.1032 | 1.2936 | 0.0164 |
| Extra Trees | 0.1413 | 0.1733 | 1.4465 | 0.004 | 0.1389 | 0.0465 | 1.2055 | 0.0272 |
| SVM | 0.1446 | -0.0541 | 1.0663 | 0.107 | 0.1444 | -0.0425 | 1.0859 | 0.1214 |
| KNN | 0.1516 | -0.2012 | 0.499 | 0.0069 | 0.151 | 0.0223 | 1.2034 | 0.0947 |
| Logistic Regression | 0.145 | -0.1221 | 0.9387 | 0.0163 | 0.1456 | -0.0596 | 1.0421 | 0.0144 |

**Table S4. DeLong test results comparing AUC between full model and reduced feature models.**

| **Model** | **Comparison** | **AUC_All** | **AUC_Reduced** | **Delta_AUC** | **Z_statistic** | **P_value** |
| --- | --- | --- | --- | --- | --- | --- |
| XGBoost | All vs Top10 | 0.8631 | 0.8667 | -0.0036 | -0.918 | 0.3588 |
| XGBoost | All vs Top5 | 0.8631 | 0.8659 | -0.0029 | -0.519 | 0.604 |
| Random Forest | All vs Top10 | 0.8659 | 0.8664 | -0.0005 | -0.127 | 0.899 |
| Random Forest | All vs Top5 | 0.8659 | 0.8557 | 0.0102 | 1.714 | 0.0866 |
| LightGBM | All vs Top10 | 0.8641 | 0.868 | -0.004 | -1.227 | 0.2199 |
| LightGBM | All vs Top5 | 0.8641 | 0.864 | 0.0001 | 0.02 | 0.9843 |
| Gradient Boosting | All vs Top10 | 0.8667 | 0.8686 | -0.002 | -0.641 | 0.5217 |
| Gradient Boosting | All vs Top5 | 0.8667 | 0.8665 | 0.0001 | 0.031 | 0.9753 |
| SVM | All vs Top10 | 0.85 | 0.8491 | 0.0009 | 0.163 | 0.8702 |
| SVM | All vs Top5 | 0.85 | 0.8536 | -0.0036 | -0.434 | 0.664 |
| Extra Trees | All vs Top10 | 0.8596 | 0.8602 | -0.0006 | -0.168 | 0.8663 |
| Extra Trees | All vs Top5 | 0.8596 | 0.8564 | 0.0032 | 0.579 | 0.5628 |
| KNN | All vs Top10 | 0.8301 | 0.8383 | -0.0082 | -0.747 | 0.4551 |
| KNN | All vs Top5 | 0.8301 | 0.8486 | -0.0184 | -1.362 | 0.1733 |
| Logistic Regression | All vs Top10 | 0.8508 | 0.8562 | -0.0054 | -1.548 | 0.1216 |
| Logistic Regression | All vs Top5 | 0.8508 | 0.8556 | -0.0047 | -0.889 | 0.3741 |

**
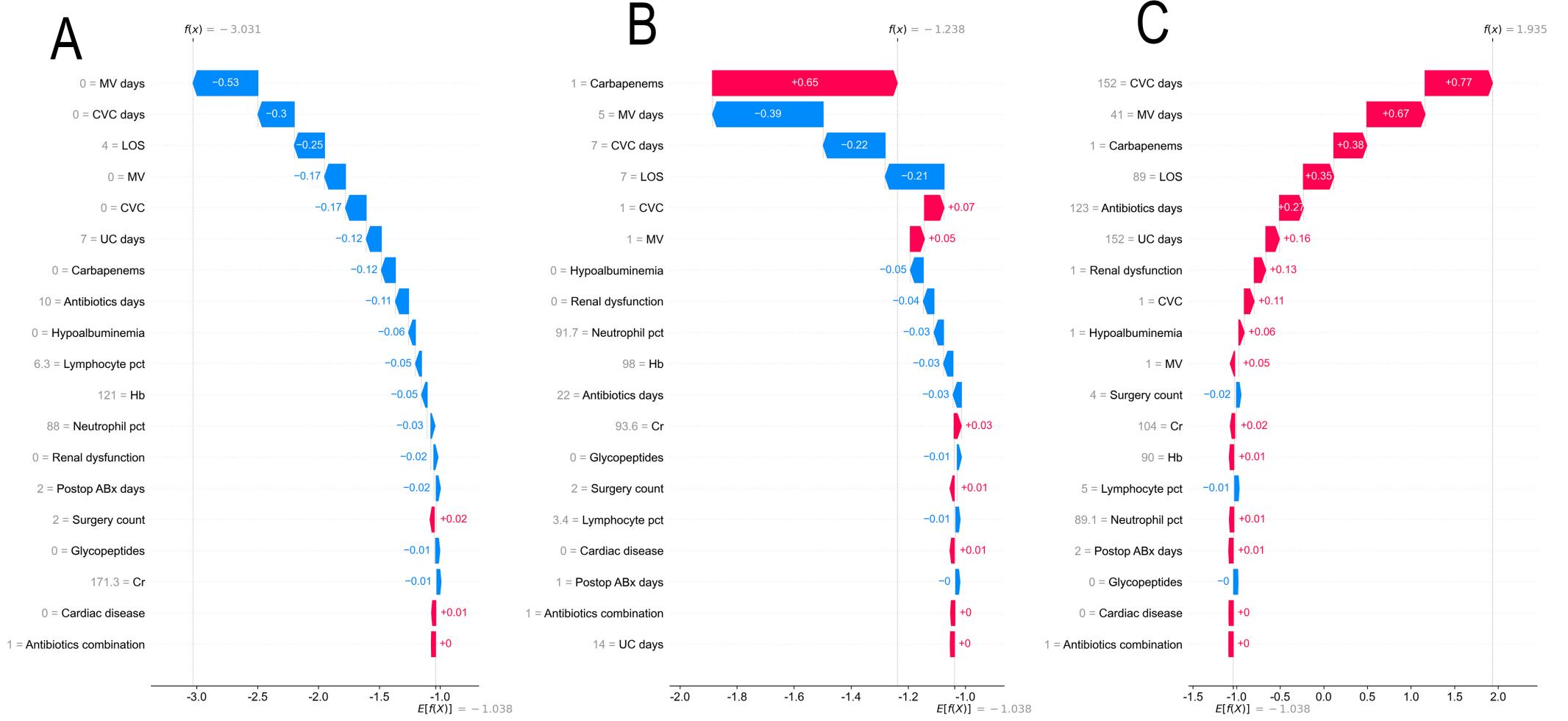
Figure S1. SHAP waterfall plots for representative patients with different risk levels.**

**
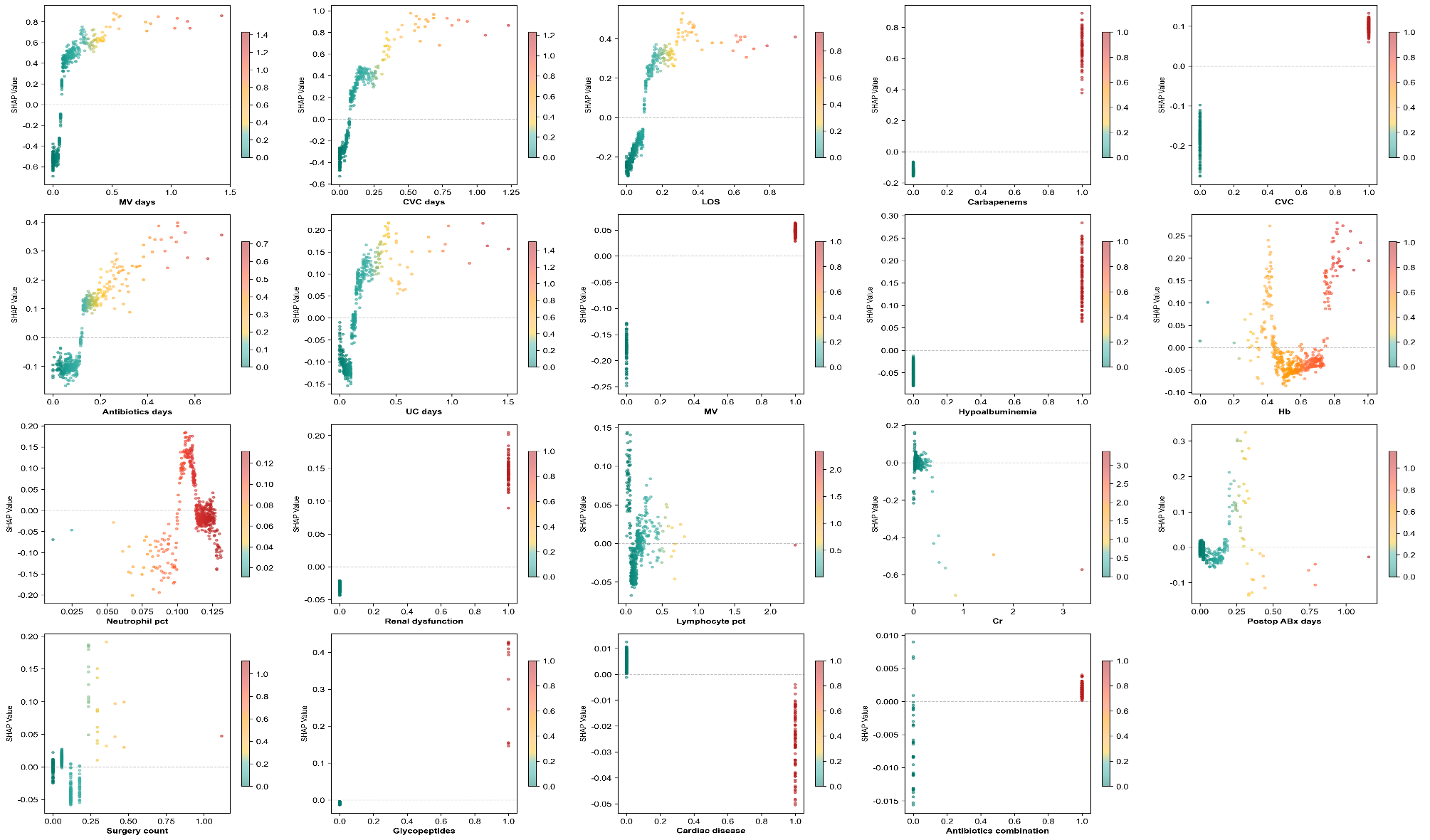
Figure S2. SHAP dependence plots for all features in the Gradient Boosting model**

**
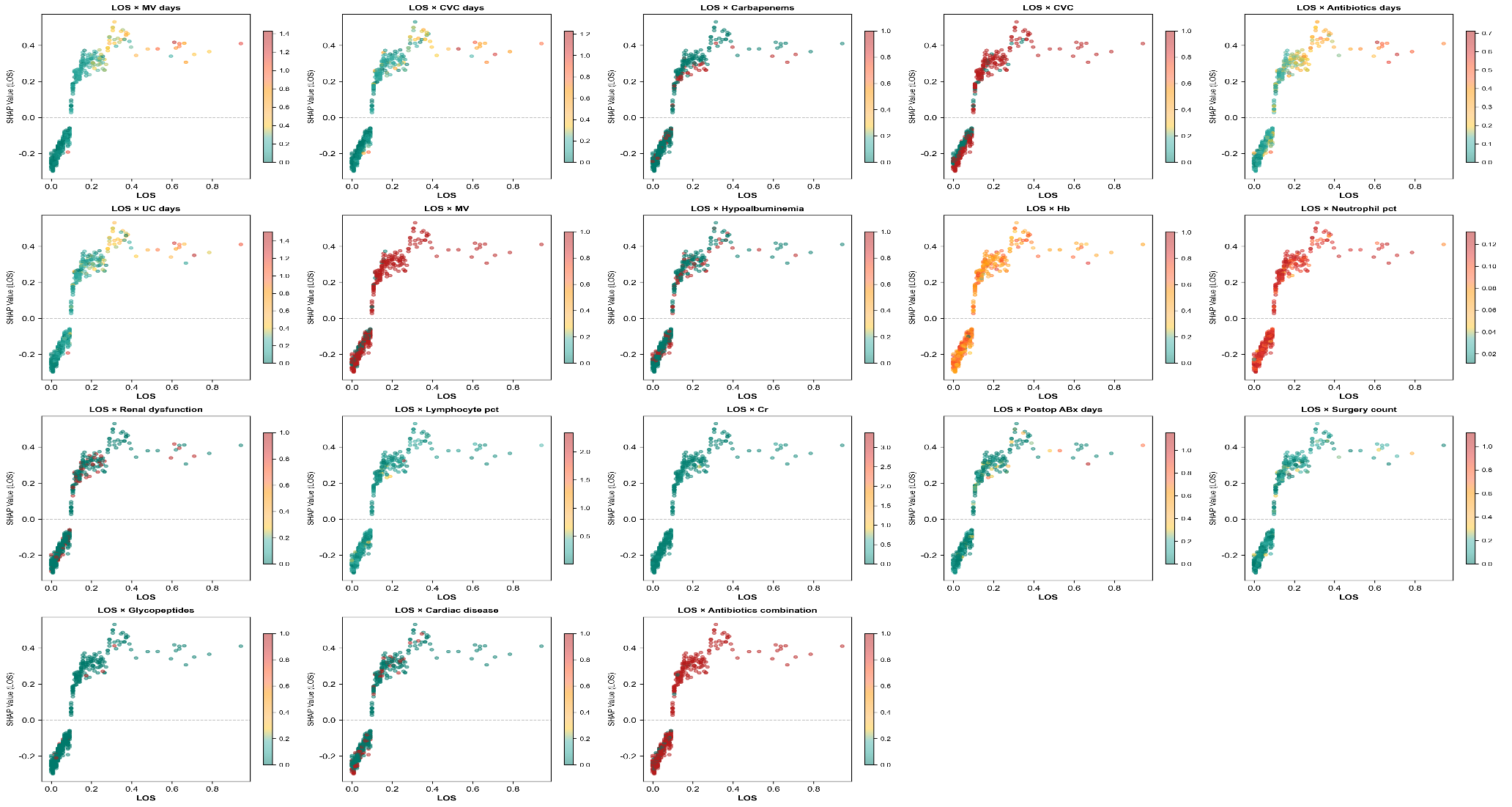
Figure S3. SHAP interaction dependence plots showing the interaction effects between ICU length of stay (LOS) and other predictive features.**

**
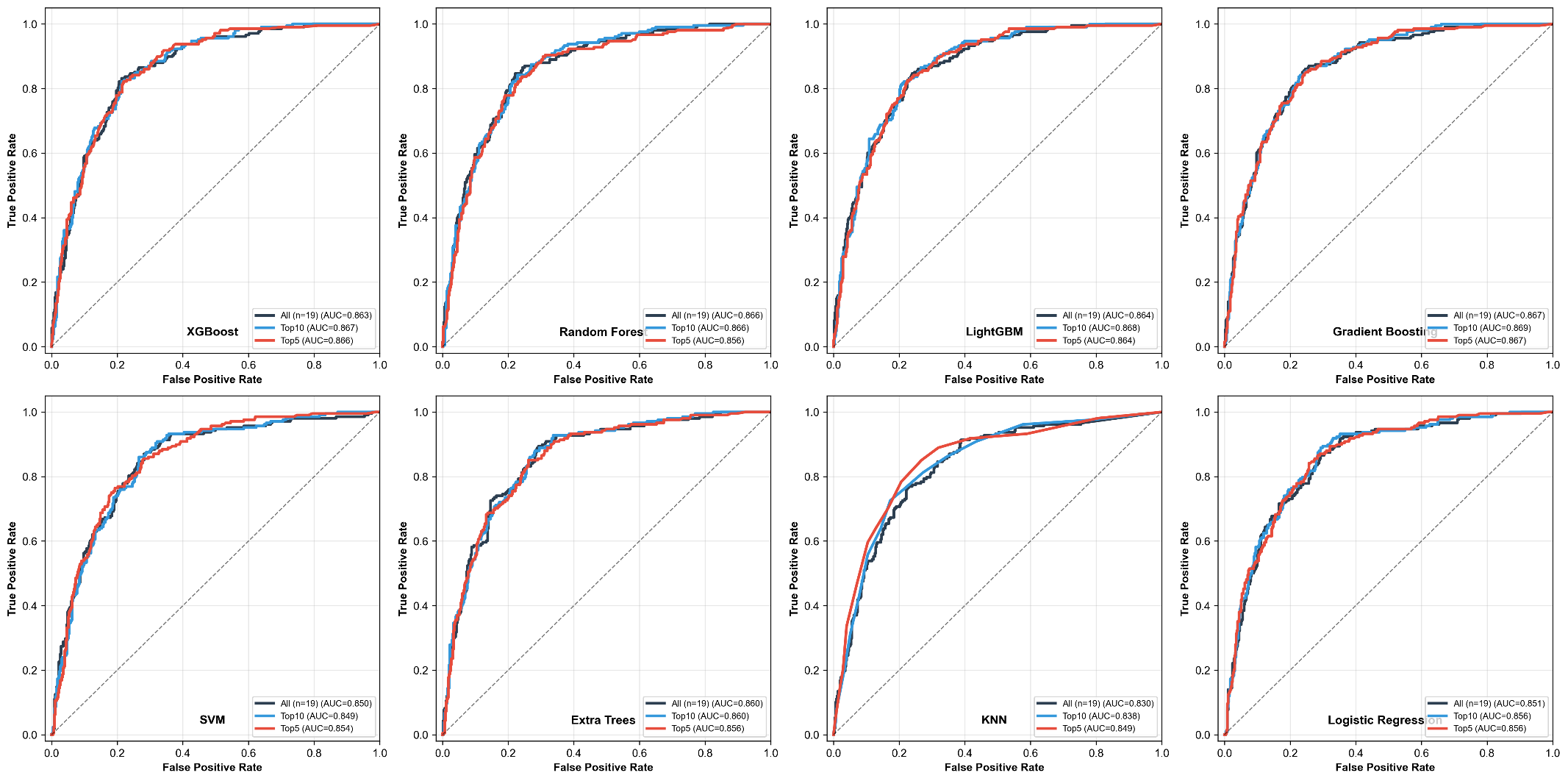
Figure S4. Sensitivity analysis: ROC curves comparing models with all features (n=19), top 10 features, and top 5 features.**
